# Supplementary material for: Risk of deep venous thrombosis associated with peripherally inserted central catheter: A retrospective cohort study of 11.588 catheters in Brazil
Source: PLoS One. 2024 May 6;19(5):e0300425. doi: 10.1371/journal.pone.0300425 (PMC11073680; doi:10.1371/journal.pone.0300425)
Supplement: Attachment — Submitted filename: Response to Reviewers.docx [file pone.0300425.s002.docx]

Additional Editor Comments:

I congratulate the authors for this valuable work. The prevalence of DVT associated with PICC and its successful implementation deserves appreciation. The manuscript was reviewed by three peer reviewers, their comments are below. A recent meta-analysis (<https://doi.org/10.1177/10760296221144041>) found that the prevalence of PICC-related DVT increased significantly with increasing catheter diameters. Do you have a method to determine the size of catheter to be used? Do you determine the catheter size based on the patient's BMI or venous diameter? Please specify.

We appreciate your comments. We have a method for determining the size of catheter to be used. Firstly, we defined the number of lumen`s catheter according to the patient’s diagnosis, comorbidities, intravenous therapy and time of hospital stay.

During the data colllection period, two equipments were used. The Site Rite 5 (BD), was used from February 2012 to January 2017. The site rite 8 (BD) started to be used from February 2017 until nowadays. This equipment provides catheter-to-vein ratio three-dimensional.

The current Infusion Nurses Society guidelines (2021) recommend the use of PICC`s with a catheter-to-vein ratio of no more than 45% prior to insertion of a vascular access device in the upper extremity, and we follow this guideline.

______________________________________________________________________

Reviewer #1: Nice and sincere efforts spent to collet these large number of cases. The draft give important idea about PICC and DVT relation. Its considered as relevant and worth data that we could depend on its results.

We appreciate your comments. We used the RedCap to collect the number of cases.

______________________________________________________________________

Reviewer #2: The authors tried to demonstrate safety and feasibility of PICC therapy introduced by a specialized team. Although it might be an interesting report, there are several issues to be revised.

We appreciate your comments to improve our study.

1) What is the main objective in this study? To analyze the risk factors for DVT or to check the competency of the special team? The conclusion does not correspond to the objective in the abstract and the manuscript as well.

Our objective is to describe the actual rate and risk factors for DVT in PICC patients in a tertiary brazilian hospital, particularly in scenario of cancer and critically-ill patients. Our hospital is accredited by Joint Commission International (JCI) and we have a service with bundles and a specialized nurses fully trainned and dedicated. We appreciate the comment about conclusion, we have rewritten this topic.

2) Why could the authors conclude that “using PICC in a service with bundles and a specialized team evidenced a low incidence of DVT”? To which factors listed in the Table 3 and 4 did the team contribute or correlate?

Some factors listed in the table 3 and 4 contributed a low incidence of DVT, such as choice of the site, type and syze of the catheter, catheter-to-vein ratio and tip location. Some factors are relation with Virchow’s triad, we had 98.2% the puncture in the first attemp, 98.2% occupancy < 33% and 98. 3% of procedures in the ideal zone, 98.3% in the superior vena cava.

3) There is little consistency and profound discussion in the Discussion section. Most paragraphs only demonstrate the data from the references. What are the new findings in this study? How do they differ from the previous studies? What is the clinical implication of the study?

We appreciate the comments about the topic discussion. Our study adds to field in a way that we sought to identify in a population with a high risk of thrombosis which were he potential risk factors associated with PICC -associated DVT. The incidence of DVT, which is a threatening complication with this catheter, is current low, provided all the best technologies and practices in insertion and maintenance are employed. We corroborate the increasing knowledge that even ICU and cancer patients, those with the highest risk for DVT might safely benefit with the use of PICC.

4) There were too many errors of English grammar and word spelling. The Conclusion section should be written in English in the 1st page. The authors should get English proofreading again for the manuscript.

We apologize for the errors of English gramar and words. We revised the manuscript.

5) Moreover, the authors should check “Submission Guidelines” of this Journal again. The title page is necessary and References should be listed according to the PLOSONE guidelines. Reviewer #2: The authors tried to demonstrate safety and feasibility of PICC therapy introduced by a specialized team. Although it might be an interesting report, there are several issues to be revised.

We attach the tittle page, and we listed the references according to the PLOSONE.

______________________________________________________________________

Reviewer #3: I think the analysis results based on the large number of cases are excellent in this paper. This paper is worthy of publication. I would like to ask you a few questions below.

We appreciate your questions.

1. What kind of treatment is positive pressure (flush) successfully performed?

We use positive pressure in polyurethane catheters. This catheter has greater rigidity, chemical, low thrombogenicity and thermosensitive. Sometimes this catheters needs to be repositioned, for example, when on the day of insertion, the catheter is located in superior vena cava and after a few days it migrated to the subclavian vein, this is not the ideal position, in this cases we use positive pressure to reposition the catheter in the vena cava superior.

2. Depending on the guidelines, patients with a history of thrombosis may be given anticoagulants, which may be beneficial in preventing thrombosis. How often do they experiencie bleeding?

Our database did not allow us to separate between the use of prophylactic and therapeutic resistance anticoagulants, although based on our hospital historical epidemiologic data, it is highly suggested that most of the anticoagulants were prophylactic. The kit to insert the catheter is with micropuncture. The adverse event bleeding ahs a low incidence in our hospital.

3. If PICC is indicated for a patient with cancer and a history of thrombosis, anticoagulants will eventually be indicated. It was important to take into account which anticoagulants were indicated or PICC were introduced, these treatments were depended on the degree of progression of the cancer. Medical systems differ in many countries, which do you prefer anticoagulants or use of PICC in your country?

Our database did not allow us to separate between the use of prophylactic and therapeutic anticoagulants, although based on our hospital historical epidemiologic data, it is highly suggested that most of the anticoagulants were prophylactic (either low molecular weight or unfractioned heparina) based on a JCI approved institution-based DVT prophylaxis guideline), with a very low number of the patients receiving oral anticoagulants..

4. This underlined part, death 11.4%) in the results of abstract, is incorrect.

Thank you for the importante observation, we have corrected this part.
